# Supplementary material for: FERN – a Java framework for stochastic simulation and evaluation of reaction networks
Source: BMC Bioinformatics. 2008 Aug 29;9:356. doi: 10.1186/1471-2105-9-356 (PMC2553347; doi:10.1186/1471-2105-9-356)
Supplement: Additional file 1 — FERN distribution, Version 1.3. This archive contains the FERN source code and binaries as well as documentation and example models in FernML and SBML. [file 1471-2105-9-356-S1.zip › fern/doc/javadoc/fern/simulation/observer/TriggerObserver.html]

TriggerObserver


---


|  |  |  |
| --- | --- | --- |
| **Overview** \| **Package** \| **Class** \| **Use** \| **Tree** \| **Deprecated** \| **Index** \| **Help** | |  |
| **PREV CLASS**   NEXT CLASS | **FRAMES**    **NO FRAMES**     **All Classes** |  |
| SUMMARY: NESTED \| FIELD \| CONSTR \| METHOD | DETAIL: FIELD \| CONSTR \| METHOD |  |


---


## fern.simulation.observer Class TriggerObserver

```
java.lang.Object
  fern.simulation.observer.Observer
      fern.simulation.observer.TriggerObserver
```

**Direct Known Subclasses:**: SBMLEventHandlerObserver

---

``` public abstract class TriggerObserver extends Observer ```

---

**Constructor Summary**

`TriggerObserver(Simulator sim)`


| **Method Summary** | |
| --- | --- |
| `abstract  boolean` | `trigger()`             Is executed, whenever a event can trigger (before step and during a theta event). |

**Methods inherited from class fern.simulation.observer.Observer**

`activateReaction, applyLabelFormat, finished, getLabel, getLabelFormat, getNumSimulations, getPrintWriter, getSimulator, getTheta, getTitlesCommand, print, setLabelFormat, setPrintWriter, setSimulator, setTheta, started, step, theta`

**Methods inherited from class java.lang.Object**

`clone, equals, finalize, getClass, hashCode, notify, notifyAll, toString, wait, wait, wait`

**Constructor Detail**

### TriggerObserver

```
public TriggerObserver(Simulator sim)
```


**Method Detail**

### trigger

```
public abstract boolean trigger()
```

:   Is executed, whenever a event can trigger (before step and during a theta event).

    :   **Returns:**: whether or not the trigger has fired


---


|  |  |  |
| --- | --- | --- |
| **Overview** \| **Package** \| **Class** \| **Use** \| **Tree** \| **Deprecated** \| **Index** \| **Help** | |  |
| **PREV CLASS**   NEXT CLASS | **FRAMES**    **NO FRAMES**     **All Classes** |  |
| SUMMARY: NESTED \| FIELD \| CONSTR \| METHOD | DETAIL: FIELD \| CONSTR \| METHOD |  |


---
